# Custom R *Flexdashboard* for molecular genetic pathology quality tracking

**DOI:** 10.1016/j.jpi.2025.100434

**Published:** 2025-03-03

**Authors:** Steven Shen, Ju-Yoon Yoon

**Affiliations:** a Department of Laboratory Medicine and Pathobiology, University of Toronto, Toronto, Ontario, Canada; b Department of Laboratory Medicine, Division of Anatomic Pathology, St. Michael's Hospital, Unity Health Toronto, Toronto, Ontario, Canada

**Keywords:** Dashboard, Quality metrics, Interactive, R, Molecular genetic pathology

## Abstract

The practice of modern-day laboratory medicine entails extensive, daily practice of tracking various quality metrics of every molecular test to ensure quality maintenance, as well as for laboratory management. While various third-party tools are commercially available, such represent a significant investment for publicly funded institutions. To automate aspects of this quality management, we developed a custom dashboard, written using R. We used R Studio, a freely available software, and employed the *Shiny* and *Flexdashboard* packages to develop the code base for the dashboard. Data for the dashboard were pulled from multiple Excel tracking spreadsheets for different clinical assays. The current dashboard allows for dynamic, automated reporting of case volume, and turn-around time, which are regularly reported metrics to CancerCare Ontario for reimbursement purposes. Workload tracking is also made possible, automating calculations regularly performed for billing purposes. The dashboard summarizes various quality metrics for each assay in a single table, viewable by multiple personnel within a single network. Additional features such as filtering quality metrics by date and customization of a variety of plots were also included. Whereas other informatics solutions may be available, our custom solution represents a low-cost system that alleviates a significant workload from various members of the laboratory medicine department, easing the currently significant administrative burden from the “hands-on” staff. Future work will be focused on further improving the accessibility of the dashboard and the integration of additional molecular assays for quality monitoring.

## Introduction

The modern laboratory medicine is increasingly becoming more complex where molecular testing is becoming an essential part of the work-up for many patients. Multiple medical disciplines such as surgery and oncology may request molecular testing to help define treatment strategies for individual patients.^1^^,^^2^ The laboratory consistently tracks a wide range of quality metrics for every molecular test on a daily basis to ensure effective quality and laboratory management. Metrics such as case volume, turn-around time (TAT), and workload calculation, are part of routine laboratory management crucial for billing, staff hiring, and financial reimbursement from government entities, among others. Digital health intervention (DHI) defined as “discrete functionality of digital technology that is applied to achieve health objectives” by the World Health Organization offers a solution to reduce the significant burden. Key performance indicator (KPI), a form of DHI, plays an important role in the evaluation, measurement, and improvement of laboratory performance.^3^ Whereas various KPI modules are commercially available, they represent a significant investment for health institutions, especially for publicly funded institutions. Furthermore, it is difficult for the standardized commercially available KPIs to cater to the specific needs of institutions. Dashboards, a type of KPI module, enable visualization of summary statistics and data and have long been utilized in various aspects of healthcare, including public health and clinical laboratories, especially during the pandemic.^4^^,^^5^ In addition, real-time dashboards for managing pathology process have shown to be effective for managers to identify bottlenecks and delays.^6^ In the province of Ontario, Canada, as part of a publicly funded healthcare system, the administrative team performed multiple data pull/tally to report various metrics to Ontario Health, and this represented a crucial step in the institution's reimbursement workflow.

While platforms such as Epic boasts dynamic avenues for KPI reporting, such solutions are platform-specific. Even when the platform is in use, the needed skillsets to build the appropriate workbench reports may not be available at the institution. Widely used spreadsheet programs such as Microsoft Excel thus remain popular in clinical laboratories for various quality tracking uses. While such programs generally allow for a solution with little-to-no additional costs, disadvantages include challenges in maintaining data fidelity with multiple persons editing the file; values inadvertently deleted are difficult to restore. Dynamic visualization is also limited, and the application of statistics can be difficult. Another advantage of spreadsheet programs is the ease of manipulation in R, an open-source, free programming language software, or similar programming languages such as Python. With the goal of automating aspects of this quality management, we aim to develop a custom written real-time dashboard written using R. The dashboard integrates various data from multiple spreadsheet files to provide a comprehensive view of laboratory metrics, enabling more efficient tracking and management. By automating data collection and analysis, we expect to improve accuracy, reduce the manual workload, and enhance decision-making processes. Lastly, dashboard was designed for multi-site institutions in mind, and thus designed to be accessible across geographically separated institution sites.

## Materials and methods

### Clinically curated data

In our clinical laboratory, specimen management and assay quality metrics tracking are performed using assay-specific tracking sheets, formulated as relational database, in form of spreadsheet files (.XLSX format). For each of the spreadsheet files, each row represents a specimen/test, with numerous associated columns, detailing aspects of specimen, and assay workflows. For all assays, the tracking sheets contain data with respect to identifiers (assay-specific identifier, and associated surgical pathology identifier), date-stamped tracking points (date collected, ordered, received, results submitted, and signed-out), time-stamped tracking points (time received). DNA extraction data, where applicable, including DNA concentrations. Assay-specific results include PCR read-out values (such as number of copies for target and reference (housekeeping) gene(s)), fluorescent in situ hybridization (FISH)-specific data (such as number of signals corresponding to *ERBB2* and CEP17), and various next generations sequencing (NGS) data (average targeted read depth, mean insert size, duplicate %, etc.).

### Developmental overview

**Fig. 1:**
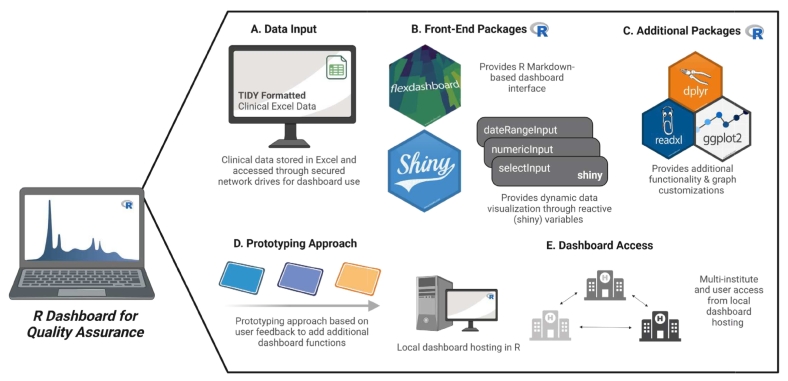
Developmental overview of a custom R dashboard for molecular genetics pathology quality tracking. The R dashboard extracts tidy data (each variable stored in its own column, and each row represents a single data observation) stored in clinical Excel tracking sheets curated and stored in network drives. The dashboard interface is built using the flexdashboard package in R Markdown providing flexible and easy customization of a dashboard interface. The shiny package is essential in providing reactive variables (date, numeric, and inputs) to drive dynamic data visualization that is responsive to filtering. Additional R packages such as readxl, dplyr, and ggplot2 provides additional functionality to read Excel tracking sheets and build customized graphs. The overall dashboard development followed a prototyping approach where user feedbacks were essential in adding additional functions and improving specific functionalities. Lastly, the dashboard is hosted locally in R and accessed across institutes for quality assurance.

We used R (version 4.3.0), a programming language for statistical computing and data visualization along with the important packages such as *Flexdashboard* (version 0.6.1)^7^ and *Shiny* (version 1.7.4)^8^ to develop the code base for the dashboard. Data for the dashboard were pulled from multiple Excel tracking spreadsheets for different clinical assays. The *Flexdashboard* package acted as the building blocks for the static dashboard interface, allowing us to include various htmlwidgets^9^ such as gauges, value boxes, grid graphics, and tabular data that are essential for the final dashboard by writing the respective codes in the R Markdown file. The *Shiny* package enabled the interactive dashboard interface, allowing the dynamic visualization of various quality metrics. Additional packages including *dpylr* (version 1.1.2),^10^ *readxl* (version 1.4.2),^11^ *ggplot2* (version 3.4.2),^12^ *plotly* (version 4.10.2),^13^ *htmltools* (version 0.5.5),^14^ and *DT* (version 0.28)^15^ were also included for the functionality of the real-time dashboard. The dashboard was developed using a prototyping approach based on user feedback. Based on user feedback, additional metrics and visualization methods were included in the final version of the dashboard (Fig. 1). The dashboard script is publicly available on GitHub (https://github.com/yoonjuy/dashboard).

### Specific dashboard functions

The dashboard was built in an R Markdown file to allow the creation of a fully reproducible workflow. Chunks imported various R packages including *flexdashboard*,^7^ *shiny*,^8^ *tidyverse*,^16^ *plotly*,^13^ *htmltools*,^14^ *DT*,^15^ *readxl*,^11^ and *anytime.*^17^ Afterwards, the multiple .xlsx clinical assay tracking sheets were imported using the *readxl* package, ensuring that each variable data type is correctly imported.

While*flexdashboard* acted as the overall base for the static dashboard, the interactive components were incorporated through the *shiny* package. The three main classes of interactivity (*dataRangeInput*, *selectInput*, and *numericInput*) can be incorporated to control various graphs, tables, and computed values. *dateRangeInput* allowed the selection of a specific date range to filter clinical data. *selectinput* allowed the selection of specific variables of interest for quality assurance to make customized graphs such as boxplots andhistograms. Selecting data fields of interest also allowed exclusion of unnecessary patient-identifying data fields, where applicable (such as medical record number). Furthermore, *numericInput* allowed further filtering of specific values within quantitative variables for further customization. An overview quality assurance summary table for various assays was generated to be filterable by date.

The specific graphs themselves were generated using the *plotly* package to allow the creation of dynamic plots responsive to data filtering from the Shiny interface. Specifically, to allow interactivity, the Shiny variables (variables that are responsive to filtering) were directly incorporated into the codes for the various plots. Additionally, filtering by date and numbers were also directly incorporated in the codes through the *filter* function of the *dpylr*^10^ package. Lastly, a tabular display of assay data points was generated using the *DT* package,^15^ where a HTML widget was used to display R data objects.

## Results

### Custom flexhdashboard for quality metrics tracking

**Fig. 2:**
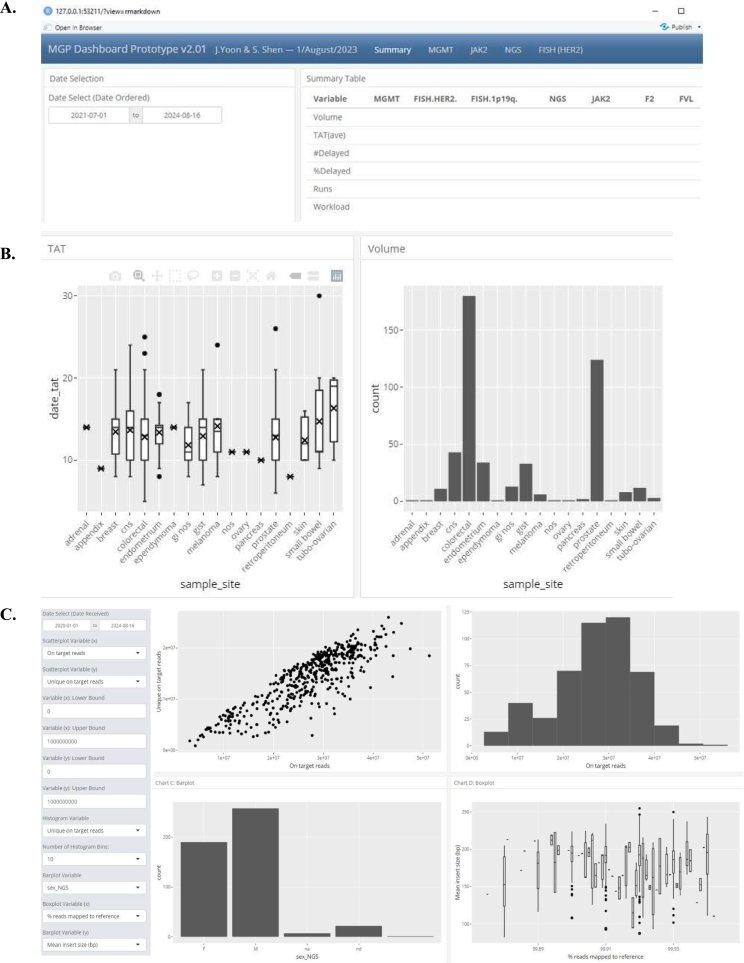
**(**A) Summary table of quality assurance metrics for various clinical assays. Note that the actual values reported have been deleted in the figure. (B) Graphical and dynamic tracking of turn-around time (TAT, date_tat) and case volume of based on specific sample site for the NGS clinical assay. (C) Customizable graphs for the cancer NGS assay-specific quality tracking were leveraged by a set of charts that can be dynamically altered. Displayed metrics include unique on-target reads (total number of reads mapping to the genomic regions covered by the custom NGS assay, minus duplicate reads), on-target reads (total number of reads mapping to the genomic regions covered by the custom NGS assay), sex_NGS = patient sex as determined by NGS (based on X chromosome coverage), and man insert size (in base pairs).

A summary table of various quality metrics such as case volume, TAT, and number of delayed cases were included in the summary table for the molecular pathology division's test menu that spans different FISH assays, PCR-based assays, and NGS panel (Fig. 2A). Additional filtering by date can be applied by selecting a specific date range on the left-hand menu, which interactively changes the resulting quality metric information on the summary table (Fig. 2B). Dynamic graphical display of quality metrics such as TAT and case volume based on specific sample sites for assays such as NGS was also included, as Ontario Health required site-specific metrics to be reported. Additional dynamic and customizable graphs such as scatterplot, histogram, barplot, and boxplot for the assays such as NGS was implemented (Fig. 2C). The left-hand menu represents the interactive component, allowing the selection of date ranges, specific continuous/categorical variables, and upper/lower limits of continuous variables for the variety of plots (Fig. 2C). Lastly, a HTML widget data table for assays such as NGS was also implemented. This dynamic table, utilizing the *DT* package in R, also allowed specific filtering of each variable and searching specific data points.

### Use in a clinical laboratory, including multi-site access

Unity Health Toronto comprises three geographically separated sites within the city of Toronto, with sites that share access to quality tracking sheets across the sites through network drives. This was made possible by locally hosting the flexdashboard at a designated workstation at the St. Michael's Hospital, where the R script was maintained, and designating an IP address. The dashboard was viewable at any of the networked stations to users with the IP address.

Since its launch, the dashboard is being actively used for regular tracking of key KPI, especially TAT. Quarterly reporting to Ontario Health, as well as to the hospital, has been in use to report two required metrics—test volume and average TAT. The dashboard was particularly helpful for the NGS assay; whereas we use a single NGS assay for all solid tumor testing, Ontario Health require us to provide the metrics for each of the disease site (e.g., colorectal, endometrium, etc.). With the disease site being one of the data fields for the NGS assay, the dashboard allowed us to quickly obtain disease site-specific key metrics. As well, disease site-specific quality metrics monitoring could be performed, allowing us to visit potential differences in the process upstream of the molecular laboratory, i.e., sample processing in the anatomic pathology division.

## Discussion

Platforms such as Epic Beaker store the various performance-related data, with numerous data points stored as discrete data variables for easier manipulation. However, such platforms require one of three solutions: (1) building of custom complex dashboard within the Epic Beaker system (e.g., Workbench Report in Cogito); (2) exporting the data through Caboodle/Clarity modules for analysis outside of the Epic Beaker system; and/or (3) an interfaced solution constructed by another vendor. Thus, even for institutions that leverage platforms such as Epic Beaker, quality data monitoring often presents a challenge, and thus more readily available solutions that use spreadsheet programs remain attractive. Of note, as our institution recently migrated to Epic Beaker, current work focuses on use of Cogito modules to pull the data in a format to be compatible with the dashboard script.

The major disadvantage of custom dashboards is the need for R scripting knowledge. Scripting and troubleshooting a custom flexdashboard R script is not necessarily a skillset readily available to laboratory technologists or molecular pathologists. Unlike some of simpler R scripts, errors may not be readily apparent in scripts that utilize flexdashboard, where launching the dashboard may only result in an ambiguous error message. From our experience, the most common type of errors were related to discordance between the data expected by the script and the data in the various tracking spreadsheets—e.g., date entered in a wrong format, to be recognized as text data. Such scenarios may also arose when laboratory technologists inadvertently changed the number of columns containing data. To address these data fidelity issues, all entered data are checked at the time of case sign-out, and the technologists were educated to improve conformance.

Another weakness of the setup is with respect to controlling access. As long as the R script is running on a host machine within the network, in the present form, any users with access to the institution's network and the IP address, can access the dashboard. Whereas the dashboard itself, in essence, allows for (enhanced) “view-only” mode, where the identifiers data displayable do not directly identify patients, controlled and monitored access may be needed at larger institutions. Also, the single hosting station represents a single failure point in the system. Whereas we maintain the RMD code on a backed-up network server, thus opening the possibility of migration, migration risks the failure related to potential changes in the R environment. Going forward, code maintenance on repositories, such as GitHub, and use of Docker containers may be employed to ensure stable environment, across workstations. Future work is to improve these aspects of the platform, including access control/logging.

Despite the above limitations and disadvantages, the advantages are obvious. By building different types of graphs into the statistics dashboard, we facilitated rapid analyses of data, while maintaining the data fidelity. Customizability of the flexdashboard with several available functions (Table 1) suitable for any quality metrics-related analyses. As well, ability to allow multi-site network access was a particularly helpful function that allowed operations lead and managers from other sites to track quality metrics from other sites. Another advantage is in consistent data analysis. Quality metrics analyses can result in heterogeneous results, based on filtering criteria employed. By scripting and thus codifying the analyses, the standard operating procedure is established for retrieval, filtering, and analysis of different quality metrics. This is a particularly useful function for laboratories where quality metrics are required to be reported to government bodies, among other recipients.

**Table 1:** Summary of functions available in the custom dashboard written in R.

Function	Description
Dynamic tabular data	Displaying a summary of data in the form of a table where variables can be filtered, and specific values can be searched
Real-time reporting	Real-time reporting of data from the multiple Excel tracking spreadsheets
Filtering	Filtering data by date and specific values are present in all quality metric summary, table, and plots
Dynamic data visualization	Displaying a summary of data in a variety of interactive & customizable plot that can be filtered
User customization	Customization of additional assays and quality metrics by direct editing in R
Workload planning	Historical quality metrics aim to inform future workload planning

## Funding source

None to acknowledge.

## Declaration of competing interest

The authors declare the following financial interests/personal relationships which may be considered as potential competing interests:

Ju-Yoon Yoon reports a relationship with Amgen Canada Inc. that includes: funding grants and speaking and lecture fees. Ju-Yoon Yoon reports a relationship with Bayer Corporation that includes: funding grants. Ju-Yoon Yoon reports a relationship with AstraZeneca R&D Reims that includes: funding grants. Ju-Yoon Yoon reports a relationship with Roche that includes: consulting or advisory. Ju-Yoon Yoon reports a relationship with Merck Sharp & Dohme Corp that includes: funding grants. Ju-Yoon Yoon reports a relationship with Pfizer that includes: funding grants. If there are other authors, they declare that they have no known competing financial interests or personal relationships that could have appeared to influence the work reported in this article.
